# Mitochondrial Transfer and Regulators of Mesenchymal Stromal Cell Function and Therapeutic Efficacy

**DOI:** 10.3389/fcell.2020.603292

**Published:** 2020-12-07

**Authors:** Amina Mohammadalipour, Sandeep P. Dumbali, Pamela L. Wenzel

**Affiliations:** ^1^Department of Integrative Biology & Pharmacology, McGovern Medical School, University of Texas Health Science Center at Houston, Houston, TX, United States; ^2^Center for Stem Cell and Regenerative Medicine, The Brown Foundation Institute of Molecular Medicine, University of Texas Health Science Center at Houston, Houston, TX, United States; ^3^Immunology Program, MD Anderson Cancer Center UTHealth Graduate School of Biomedical Sciences, Houston, TX, United States

**Keywords:** cancer metabolism, hematological malignancy, mesenchymal stromal cells (MSCs), MSC differentiation, metabolic reprogramming, mitochondrial biogenesis, mitochondrial dynamics, mitochondrial transfer

## Abstract

Mesenchymal stromal cell (MSC) metabolism plays a crucial role in the surrounding microenvironment in both normal physiology and pathological conditions. While MSCs predominantly utilize glycolysis in their native hypoxic niche within the bone marrow, new evidence reveals the importance of upregulation in mitochondrial activity in MSC function and differentiation. Mitochondria and mitochondrial regulators such as sirtuins play key roles in MSC homeostasis and differentiation into mature lineages of the bone and hematopoietic niche, including osteoblasts and adipocytes. The metabolic state of MSCs represents a fine balance between the intrinsic needs of the cellular state and constraints imposed by extrinsic conditions. In the context of injury and inflammation, MSCs respond to reactive oxygen species (ROS) and damage-associated molecular patterns (DAMPs), such as damaged mitochondria and mitochondrial products, by donation of their mitochondria to injured cells. Through intercellular mitochondria trafficking, modulation of ROS, and modification of nutrient utilization, endogenous MSCs and MSC therapies are believed to exert protective effects by regulation of cellular metabolism in injured tissues. Similarly, these same mechanisms can be hijacked in malignancy whereby transfer of mitochondria and/or mitochondrial DNA (mtDNA) to cancer cells increases mitochondrial content and enhances oxidative phosphorylation (OXPHOS) to favor proliferation and invasion. The role of MSCs in tumor initiation, growth, and resistance to treatment is debated, but their ability to modify cancer cell metabolism and the metabolic environment suggests that MSCs are centrally poised to alter malignancy. In this review, we describe emerging evidence for adaptations in MSC bioenergetics that orchestrate developmental fate decisions and contribute to cancer progression. We discuss evidence and potential strategies for therapeutic targeting of MSC mitochondria in regenerative medicine and tissue repair. Lastly, we highlight recent progress in understanding the contribution of MSCs to metabolic reprogramming of malignancies and how these alterations can promote immunosuppression and chemoresistance. Better understanding the role of metabolic reprogramming by MSCs in tissue repair and cancer progression promises to broaden treatment options in regenerative medicine and clinical oncology.

## Introduction

MSCs are multipotent non-hematopoietic cell precursors found in the bone marrow. Albeit highly heterogeneous, similar multipotent stromal cells are ubiquitous in many other tissues throughout the body, such as connective tissue and tumor stroma, and are also referred to as MSCs in the literature (Ullah et al., 2015). MSCs are classical mesodermal derivatives that give rise to chondrocytes, adipocytes, and osteoblasts but are also purported to activate genes required for specialized cells of the ectoderm and endoderm. MSCs have attracted great clinical interest for their ability to differentiate into various cell types, secrete trophic and immunomodulatory factors, and sense and respond to signals generated by inflammation, tissue injury, or tumorigenesis, such as damage associated molecular patterns (DAMPs) (Garg et al., 2010; Krysko et al., 2011). Substantial evidence now suggests that mitochondria play crucial roles as signaling molecules to MSCs and that mitochondrial biogenesis and quality control is vital for MSC function, self-renewal, and differentiation.

Mitochondria are biological powerhouses that provide cellular fuel through conversion of nutrients to energy. In order to meet the demands of cellular activity, mitochondria are responsible for generating the majority of the cell's energy–adenosine triphosphate (ATP)–through oxidative phosphorylation (OXPHOS). In addition to aerobic energy production, mitochondria also play central roles in reactive oxygen species (ROS) production, calcium homeostasis, cellular signaling, and synthesis and/or assembly of metabolites, including fatty acids, amino acids, iron/sulfur clusters, pyrimidines, heme, and steroid hormones (Seo et al., 2018). Mitochondria modulate a range of cellular functions, including apoptosis, autophagy, cell cycle regulation, differentiation, and aging (Rodriguez et al., 2018). As a result, mitochondria are important for cellular adaptation to physiological and pathological microenvironments. Indeed, dysfunction in mitochondria has been linked to cellular aging and several human diseases. Mutations in mitochondrial DNA (mtDNA) or nuclear genes encoding mitochondrial proteins can cause acquired or inherited mitochondrial disease. Less obvious is the mitochondrial dysfunction that occurs in the absence of mutations in mitochondrial genes, which appears as a result of infection, genotoxic insults, or other environmental challenges and can contribute to degenerative disease. Dysfunctional mitochondria produce unhealthy levels of ROS, such as peroxides, superoxide, hydroxyl radical, singlet oxygen, and alpha-oxygen, that damage lipids, protein, and DNA (Li C. et al., 2019). Mitochondrial dysfunction is also tied to ROS-independent factors, including impaired mitochondrial integrity and defective mitochondrial quality control. In a surprising act of altruism, healthy cells can donate or transfer their mitochondria or mtDNA to cells with aged or damaged mitochondria, thus restoring biological fitness of the recipient cell. In this regard, MSCs can exert powerful regenerative effects on aging or injured cells via transfer of mitochondrial cargo.

Despite the therapeutic role of MSCs in tissue homeostasis and repair, MSCs and other stroma can be hijacked for recruitment to tumors to contribute to cancer progression. MSCs can alter metabolism of cancer cells through modification of metabolites and/or nutrients in the microenvironment and by transfer of mitochondria or mtDNA to cancer cells. Mitochondria have diverse effects in tumorigenesis and cancer progression. While some, but not all, cancer cells are heavily dependent upon aerobic glycolysis (Warburg effect), many classical hallmarks of cancer include altered mitochondrial function that extends beyond energy production (Vyas et al., 2016). From cancer initiation to metastasis, mitochondria influence oncometabolite generation, metabolic reprogramming, oxidative signaling, redox homeostasis, cell survival, and metastatic behavior. The ability of MSCs to shelter malignant cells is clinically significant, since MSCs have been shown to reinforce the pro-glycolytic phenotype of leukemia cells and minimize ROS-induced damage to cancer cells (Samudio et al., 2008; Ohkouchi et al., 2012). Furthermore, MSCs have been found to donate mitochondria to solid tumor cells, including breast and lung cancer (Spees et al., 2006; Pasquier et al., 2013). Mitochondrial transfer is also well-documented in hematological malignancies, such as acute myeloid leukemia (AML), acute lymphocytic leukemia (ALL), and multiple myeloma (MM) (Moschoi et al., 2016; Griessinger et al., 2017; Marlein et al., 2017). In the context of cancer treatment, several studies have revealed that transfer of mitochondria from MSCs can protect mutant hematopoietic cells during chemotherapy.

In this review, we summarize recent progress in the study of mitochondria in MSC differentiation, mitochondrial trafficking mechanisms, and therapeutic applications. We specifically address the mechanisms that mediate mitochondrial transfer and biological consequences of mitochondrial transfer between MSCs and the tumor microenvironment in solid tumors and hematological malignancies.

## MSC Differentiation

The metabolic properties of cells in the bone marrow tightly correlate with their capacity to self-renew, proliferate, endure stress, perform specialized functions, and differentiate. Hematopoietic stem cells (HSCs) and MSCs both reside in the bone marrow, along with their corresponding progenitors at various states of differentiation, mature immune cells, adipocytes, osteoblasts, osteoclasts, stromal cells, endothelial cells, pericytes, and nerve cells. Communication among these cells in various regions of the marrow is crucial in maintenance of hematopoietic stem and progenitor cell homeostasis (Uccelli et al., 2008). The inner lining of the bone surface that interfaces with the marrow, or the endosteum, houses MSCs and other osteoprogenitors. There, MSCs contribute to osteogenesis by interacting with osteoblasts and local hematopoietic cells (Boroumand and Klip, 2020). The bone marrow vasculature also provides distinct microenvironments in the form of sinusoidal and arterial niches, likely characterized by different levels of oxygen tension. Both vessels are lined by endothelial cells but the sinusoid is surrounded by Leptin receptor-expressing stromal cells and arterioles are ensheathed by NG2-expressing peri-arteriolar cells, sympathetic nerves, and Schwann cells (Asada et al., 2017). These niches modulate HSC quiescence, self-renewal, and differentiation by direct interaction and release of hematopoietic growth factors and cytokines. Pathological conditions can alter the bone marrow microenvironment, leading to dysregulated differentiation and behavior of MSCs and their progeny. In addition, a substantial body of literature indicates that MSC self-renewal and differentiation not only depend on the cellular composition of the niche, but that they also rely on environmental stimuli from hormones, nutrients, and oxygen. MSCs in the bone marrow experience gradients of oxygen abundance and low oxygen tension, or hypoxia. Undifferentiated MSCs that reside in hypoxic regions of the marrow engage in anaerobic glycolysis, which is essential for survival in hypoxic environments (Mohyeldin et al., 2010). Upon differentiation of MSCs, cells must undergo a metabolic switch away from glycolysis toward oxygen- and mitochondria-dependent OXPHOS. Thus, metabolic reprogramming through mitochondrial mediators plays a central role in determining cell fate of MSCs as well as other stem cells. Mitochondrial abundance, morphology, age, activity, fuel usage, and metabolite production can all influence stem cell renewal and fate decisions (Zhang et al., 2018; Bahat and Gross, 2019). Below, we introduce the metabolic reprogramming that accompanies lineage commitment of MSCs and describe mitochondrial features and regulation that participate in this adaptation.

### Hypoxia and Metabolism

Self-renewal of MSCs is thought to be greatest in hypoxic regions of the bone marrow (Mohyeldin et al., 2010). Similarly, long-term plasticity and expansion of *ex vivo* cultured MSCs is improved under physiological conditions of 2–5% oxygen (Grayson et al., 2006; Boyette et al., 2014). Adaptation to low oxygen environments is mediated in part by hypoxia-inducible factor-1α (HIF-1α), a transcription factor that is stabilized by low oxygen tension (Semenza, 1998). In contrast, high oxygen tension typical of normoxic conditions (20% oxygen) accelerates proteolytic degradation of HIF-1α, thereby reducing total HIF-1α levels in the cell. HIF-1α has been shown to play an essential role in maintenance of MSC stemness and inhibition of terminal differentiation under hypoxia (Yun et al., 2002; Lin et al., 2006). Differentiating MSCs typically undergo a dramatic decrease in glycolysis, concurrent with enhanced mitochondrial respiration (Hofmann et al., 2012; Hsu et al., 2016). HIF-1α constrains this metabolic reprogramming through transactivation of genes required for anaerobic glycolysis while also suppressing genes necessary for mitochondrial respiration (Semenza, 1998; Kondoh et al., 2007). Thus, HIF-1α stabilization in low oxygen environments aids in preservation of MSC stemness via inhibition of the metabolic shift to OXPHOS.

Evidence in the literature supports a role for oxygen tension in determination of MSC fate and lineage potential. For example, bone marrow-derived MSCs in three-dimensional (3D) pellet cultures showed the ability to undergo enhanced chondrogenic differentiation in hypoxic conditions, as evidenced by upregulation of cartilage matrix genes and chondrogenesis-associated genes such as the transcription factor SOX6 (Khan et al., 2010). Moreover, fate selection is greatly influenced by changes in oxygen tension. Following normoxic expansion of MSCs, hypoxia amplifies osteogenesis-associated genes, elevates mineral deposition, and enhances chondrogenesis in 3D pellet cultures, while normoxia inhibits adipogenesis (Boyette et al., 2014). Indeed, severe hypoxia elevates osteoblast lineage-specific transcripts, such as ALPL, the gene that encodes the alkaline phosphatase enzyme important for bone mineralization (Ejtehadifar et al., 2015). Conversely, differentiating MSCs in normoxic conditions express increased levels of adipogenic transcripts (Boyette et al., 2014). In further support of a role for hypoxia in MSC lineage commitment, HIF-1α knockdown suppresses hypoxia-induced osteogenesis (Wagegg et al., 2012). It is important to note, however, that hypoxia alone is not sufficient to induce expression of all osteoblast-specific transcripts, such as RUNX2, highlighting the importance of other soluble instructive cues in lineage maturation. HIF-1α has also been shown to be essential for chondrocyte differentiation and survival in physiological hypoxic environments and controls a complex homeostatic response during cartilage and bone development (Araldi and Schipani, 2010).

### Mitochondrial Biogenesis

**Figure 1:**
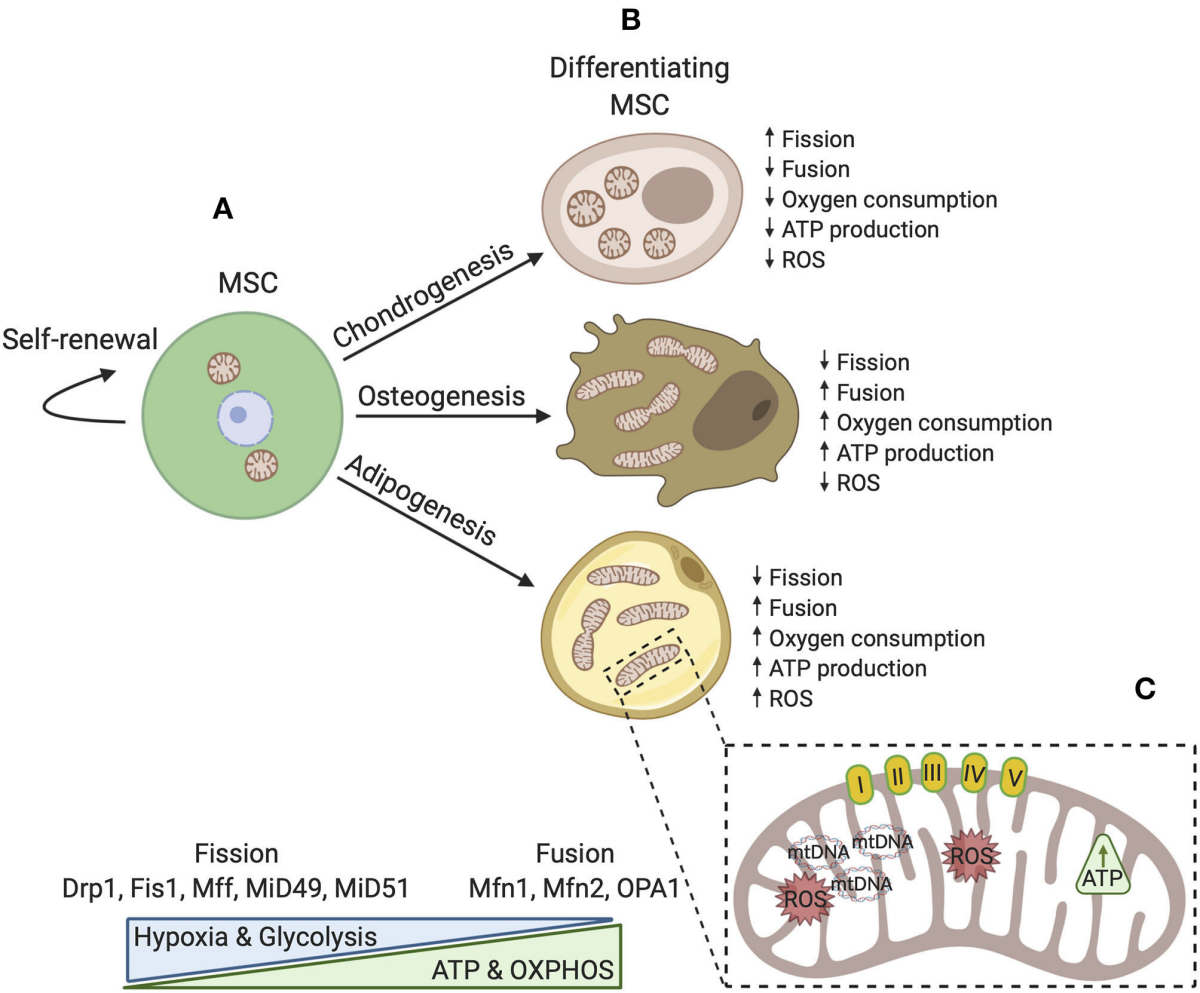
A simplified schematic model of mitochondrial dynamics in MSCs during self-renewal and differentiation. **(A)** In the undifferentiated state, mitochondrial morphology is fragmented with few cristae localized around the nucleus. Fission proteins maintain a fragmented mitochondrial network where glycolysis is a major source of energy production. OXPHOS, intracellular ATP, and ROS levels are low. **(B)** During differentiation, mitochondria are generally distributed throughout the cytoplasm and are reorganized. Distinct differences exist dependent upon cell fate such that osteoblasts and adipocytes favor development of an interconnected tubular network with higher cristae density. In these cell types, mitochondrial biogenesis is activated and fusion proteins aid in maintenance of elongated mitochondria. Osteoblasts and adipocytes also rely more upon OXPHOS and generally produce more ATP. Conversely, chondrocytes possess spherical mitochondria and produce less energy by OXPHOS. **(C)** Upon differentiation, mitochondrial capacity is modified by several mechanisms to ensure that the demands of adipogenic, chondrogenic, and osteogenic lineages are met.

Bioenergetic demand and capacity evolve as cellular functions change. A striking adaptation in differentiated progeny is the increase in mitochondrial capacity and efficiency. During osteogenesis and adipogenesis, mitochondrial membrane potential, respiratory enzyme complexes, oxygen consumption, and intracellular ATP content are all elevated (Chen et al., 2008; Tahara et al., 2009; Pietilä et al., 2012). Osteogenic induction appears to also induce mitochondrial biogenesis and increase mtDNA copy number (Chen et al., 2008; Pietilä et al., 2012). Interestingly, mtDNA copy number steadily increases over the course of osteogenic maturation and enhances mitochondrial biogenesis (Chen et al., 2008). Consistent with a decreased dependence upon glycolysis, these cells also exhibit reduced lactate production. Notably, mitochondrial mass does not appear to be increased during chondrogenesis, highlighting specificity in the bioenergetic requirements of different lineages (Forni et al., 2016). Adaptations in mitochondrial abundance and dynamics in MSCs during lineage commitment are depicted in Figure 1.

Consistent with an increased reliance on OXPHOS, regulators of mitochondrial biogenesis are altered during MSC differentiation. Peroxisome proliferator-activated receptor gamma coactivator 1-alpha (PGC-1α) is a master regulator of mitochondrial biogenesis. PGC-1α and mtDNA content are both increased in human bone marrow MSCs undergoing adipogenic differentiation (Fernandez-Marcos and Auwerx, 2011; Huang et al., 2011; Hofmann et al., 2012; Zhang et al., 2013). PGC-1α knockdown inhibits adipogenesis; whereas, PGC-1α overexpression significantly increases expression of genes involved in mitochondrial functions, lipid metabolism, and brown adipose tissue commitment (Huang et al., 2011). PGC-1α activates nuclear respiratory factors (NRF1 and NRF2) which, in turn, modulate mitochondrial transcription factor A (TFAM) (Ventura-Clapier et al., 2008). Together, TFAM and DNA polymerase γ are essential for replication of the mitochondrial genome (Ekstrand et al., 2004). Upon osteogenic induction of MSCs, increased PGC-1α is accompanied by increases in TFAM and DNA polymerase γ (Chen et al., 2008). Upregulation of TFAM has also been reported during adipogenic and chondrogenic differentiation of MSCs (Chen et al., 2008; Zhang et al., 2013). Interestingly, knockdown of TFAM suppresses mitochondrial activity and blocks adipogenic differentiation of MSCs (Zhang et al., 2013). In this study, TFAM knockdown was accompanied by a significant decrease in MtND2 (TFAM-dependent mitochondrial gene), while CytC (TFAM-independent gene) remained unchanged, indicating that TFAM is a key activator of mitochondrial transcription and genome duplication in MSC lineage commitment (Zhang et al., 2013). Two pathways believed to lie upstream of PGC-1α regulation in MSCs include Wnt-MAPK signaling and the AMP-activated protein kinase (AMPK) energy and nutrient sensor pathway. In a study of Wnt-induced osteoblastic differentiation of embryonic MSCs, An et al. (2010) revealed that mitochondrial biogenesis plays an important role in the osteoblastic differentiation of mesenchymal progenitor cells. Wnt/β-catenin signaling potently induced PGC-1α, TFAM, and mitochondrial biogenesis through activation of the Erk and p38 MAPK pathways (An et al., 2010). In a second study of genetically modified MSCs not undergoing differentiation, ectopic expression of AMPK triggered upregulation of PGC-1α and TFAM (De Meester et al., 2014). From these data, we conclude that mitochondrial metabolism of differentiating and self-renewing MSCs is greatly influenced by regulation of PGC-1α, mtDNA copy number, and mitobiogenesis.

### Mitochondrial Dynamics

Mitochondria also change shape to meet cellular demands. Cycles of mitochondrial fusion and fission, motility, and cristae remodeling, collectively referred to as mitochondrial dynamics, regulate and can be affected by mitochondrial activity (Cogliati et al., 2016; Mishra and Chan, 2016). Further, deep invaginations in the inner mitochondrial membrane (IMM) known as cristae exhibit profound diversity in structure and composition (Perkins et al., 1997; Zick et al., 2009). Mitochondrial machinery, including electron transport chain complexes, surround and are embedded within the membranes of cristae and thus cristae topology influences respiratory efficiency by regulating assembly of respiratory complexes and supercomplexes, nucleoid maintenance, iron-sulfur biogenesis, and protein synthesis and translocation (Gilkerson et al., 2003; Vogel et al., 2006; Cogliati et al., 2013; Kopek et al., 2013).

Mitochondrial remodeling accompanies fate commitment of both embryonic and adult stem cells. In undifferentiated MSCs, mitochondria localize near the nucleus (perinuclear) and adopt a fragmented spherical shape (Hofmann et al., 2012; Quinn et al., 2013). As MSCs differentiate and mature, a branched network of elongated mitochondria forms uniformly throughout the cytoplasm during osteogenesis and adipogenesis (Hofmann et al., 2012; Quinn et al., 2013). Conversely, chondrogenesis is associated with fragmented spherical mitochondria (Forni et al., 2016; Seo et al., 2018). In addition, a feature unique to terminally differentiated adipocytes is mitochondrial enrichment around lipid droplets (Hofmann et al., 2012), a phenomenon that positions mitochondria closer to respiratory substrates in the lipids and is thought to be particularly relevant in times of nutrient depletion (Rambold et al., 2015). These changes in morphology and distribution occur in conjunction with a shift in metabolism from glycolytic to oxidative, resulting in higher intracellular levels of ATP and ROS (Figure 1). Careful characterization of ultrastructure remains incompletely described in MSCs, but, based upon studies of other stem cells, it is probable that cristae shape and density are also remodeled to support increased capacity for ATP synthesis via modulation of supercomplex assembly and stability (Cogliati et al., 2013).

Morphology and ultrastructure of mitochondria are controlled by numerous mitochondrial-shaping proteins, including GTP-dependent dynamin-like proteins involved in fusion and fission cycles, as well as structural proteins (Pernas and Scorrano, 2016). Mitochondrial division, or fission, requires the actin cytoskeleton and endoplasmic reticulum (ER), along with cytoplasmic partners and adaptor proteins. Mounting evidence supports a model for fission in which the actin cytoskeleton pre-constricts mitochondria at ER contact sites (Hatch et al., 2014). ER-bound inverted formin 2 (INF2) is essential for mediating actin polymerization at these sites, thus triggering a transfer of calcium ions from the ER across the outer mitochondrial membrane (OMM). Elevated matrix calcium activates IMM constriction (Chakrabarti et al., 2018). Actin polymerization also stimulates recruitment of another central player in fission, dynamin-1-like protein (Drp1). Subsequent to IMM division, Drp1 forms oligomer rings around the mitochondria to promote OMM division (Ji et al., 2015). These sites are occupied by a host of other pro-fission and adaptor proteins, including mitochondrial fission protein 1 (Fis1), mitochondrial fission factor (Mff), and mitochondrial dynamics proteins of 49 kDa and 51 kDa (MiD49 and MiD51) which also contribute to loading of Drp1 on fission sites (Losón et al., 2013) (Figure 1A). Murine dermis-derived MSCs undergoing chondrogenesis express more Drp1, Fis1, and Fis2, consistent with their fragmented mitochondrial morphology (Forni et al., 2016). Using an assay based on dilution of a mitochondrially targeted photoactivatable green fluorescent protein, Forni et al. (2016) observed that chondrogenic MSCs undergo fewer fusion events than both undifferentiated MSCs and MSCs differentiated under osteo- and adipogenic conditions. Further support for the importance of mitochondrial remodeling comes from overexpression of a dominant negative Drp1, which resulted in a reduced ability to induce mitochondrial fragmentation and decreased expression of *Col2a* and *Sox9*, two genes important for cartilage formation (Hirao et al., 2006; Eslaminejad et al., 2013; Forni et al., 2016). This suggests that reorganization of the mitochondrial network toward a fragmented spherical phenotype plays an active role in commitment of MSCs to the chondrocyte lineage.

Similarly, specification of osteocytes and adipocytes is influenced by factors that drive fusion of mitochondria. The fusion of mitochondria requires carefully orchestrated tethering of the OMM of each mitochondrion, followed by fusion of the IMMs. Several GTPase proteins participate in tethering and mixing of mitochondrial contents, including optic atrophy protein 1 (OPA1), mitofusin-1 (Mfn1), and mitofusin-2 (Mfn2) (Cipolat et al., 2004) (Figure 1B). Along with development of an interconnected tubular mitochondria network, *Mfn1* and *Mfn2* transcripts are upregulated in the early steps of osteogenesis and adipogenesis (Forni et al., 2016; Seo et al., 2018). Knockdown of *Mfn2* results in reduced mitochondrial fusion and oxygen consumption, which causes impaired differentiation (Forni et al., 2016). Specifically, *Mfn2* knockdown blocked deposition of calcified matrix and expression of genes activated upon development of preosteoblasts into mature osteoblasts, *Ost, Runx2*, and *Dlx5*. Likewise, adipocytic cultures produced far less lipid droplets and had reduced expression of genes encoding adipocyte transcription factors, peroxisome proliferation-activated receptor γ (PPARγ), Rxr, and Creb. There is also evidence to suggest that cristae morphology is critical for osteogenic fate and maintenance. Knockdown of the gene that encodes a master regulator of cristae dynamics, Mitofilin (encoded by *Immt*), which is a core component of the mitochondrial contact site and cristae-organizing system (MICOS) complex, produces giant mitochondria with high IMM to OMM ratios, low mitochondrial membrane potential, decreased ATP generation, and elevated ROS. The insufficiency in Mitofilin disrupts osteogenesis and causes weak alkaline phosphatase activity, reduces mineralization capability, and downregulates expression of osteogenic marker genes, *Runx2, Osx*, and *Ocn* (Lv et al., 2018). Collectively, these data implicate mitochondrial morphology and cristae architecture in fate specification of MSCs.

### Reactive Oxygen Species

Reactive oxygen species (ROS) are chemical species containing oxygen that readily react with organic substrates, including lipids, proteins, sugars, and nucleic acids. ROS are primarily generated from NADPH oxidase in the cytoplasmic membrane and enzyme complexes of the electron transport chain (Collin, 2019). Other sources include enzymes in the endoplasmic reticulum (xanthine oxidase, lipo- and cyclo-oxygenase, cytochrome P450) and peroxisomes (Collin, 2019).

Aging and oxidative stress have been reported to cause cellular dysfunction by impacting MSC mitochondrial dynamics (Stab et al., 2016). Long-term MSC culture results in changes in mitochondrial morphology, decreased antioxidant capabilities, and increased ROS levels (Geißler et al., 2012). A comparison between young and old MSCs in culture illustrates that aging enhances mitochondrial fusion through downregulation of Drp1 and upregulation of Mfn2 (Li X. et al., 2019). On the other hand, oxidative stress induced by ROS overgeneration results in mitochondrial fission and fragmentation in MSCs. Conversely, a combination of N-acetyl-l-cysteine (NAC) and ascorbic acid 2-phosphate reduces ROS along with inhibition of mitochondrial fission (Li et al., 2015). Similarly, oxidative stress induced by serum deprivation and hypoxia in MSCs prompts mitochondrial fission associated with upregulation of Drp1 and downregulation of Mfn2 expression (Deng et al., 2020).

Several studies have also examined the role of ROS and related signaling pathways in the regulation of MSC differentiation and senescence. Indeed, although mitochondrial activity and ROS generation are elevated during MSC differentiation, it remained unclear whether these metabolic adaptations were essential for differentiation or simply byproducts of the differentiation process. Tormos et al. (2011) tested the importance of ROS in the differentiation of adipocytes by targeting antioxidants to the mitochondria of human MSCs. They found that antioxidants inhibited adipocyte differentiation, while addition of exogenous hydrogen peroxide could rescue adipogenesis. Further, authors identified the source of the pro-adipogenic ROS as mitochondrial complex III. Ablation of complex III function through knockdown of one of its essential components, Rieske iron sulfur protein (RISP), revealed that superoxide generated by this complex is necessary to initiate adipocyte differentiation. Interestingly, treatment of MSCs with antimycin A, which diminishes oxygen consumption but permits superoxide generation, did not interfere with upregulation of adipogenic markers such as PPARγ. This pro-adipogenic effect of superoxide could have implications for expansion of adipocytes in the marrow, such as is typical during aging (Almeida and O'Brien, 2013; Hu et al., 2018), after myeloablation (Kiang, 2016), and in AML (Hole et al., 2013; Shafat et al., 2017; Sillar et al., 2019). For example, increased levels of superoxide have been reported in 65% of primary AML blasts compared to normal bone marrow samples (Hole et al., 2013). Thus, various metabolic conditions that intensify stress conditions in the bone can result in enhanced marrow adiposity. Overall, this study provides compelling evidence that ROS generation is not simply a consequence of differentiation but rather is an effector of adipocyte differentiation. Subsequent studies corroborate these conclusions. For example, Lin et al. (2018) found that lipid droplet formation in the mouse MSC line C3H10T1/2 was improved with hydrogen peroxide treatment, while calcium deposition as an indicator of osteogenesis was dramatically reduced. Consistent with the trend toward enhanced adipogenesis and repressed osteogenesis, authors showed upregulation in early and late adipogenic transcription factors KLF5, C/EBPb, PPARc2 and LEPTIN and downregulation of osteogenic transcription factors RUNX2, c-MAF and COL1A. Conversely, these effects were reversed by the ROS scavenger N-acetyl-l-cysteine (NAC).

### Sirtuins in Metabolic Reprogramming

Metabolic reprogramming of differentiating MSCs is accomplished in part by post-translational modification of proteins required for MSC maintenance and differentiation (Li et al., 2011; Xiao and Chen, 2012; Jeon et al., 2016). Post-translational modification in the mitochondria and protein-protein interactions that modify diverse mitochondrial functions are impacted by cellular demand, nutrient availability, and redox conditions. The sirtuin family of NAD^+^-dependent protein deacetylases plays a central role in this sensing and regulates cytoplasmic enzymes, chromatin state, and other proteins that dictate response. Seven mammalian sirtuins regulate transcriptional repression, recombination, cell cycle, microtubule organization, and cellular response to DNA-damaging agents (North and Verdin, 2004). SIRT1, SIRT6, and SIRT7 catalyze post-translational modification of proteins in the nucleus. SIRT3, SIRT4, and SIRT5 act chiefly on proteins within the mitochondria. SIRT2 primarily localizes to the cytosol, though it can transiently shuttle to the nucleus in a cell cycle-dependent manner. Here, we summarize the role of these sirtuins in metabolic reprogramming of MSCs.

SIRT1 is perhaps the most well-studied sirtuin. Substantial evidence supports roles for SIRT1 in energy metabolism and a variety of physiologic and pathologic events, cell survival, differentiation, and age-related alterations. In the content of aging, activation of SIRT1 by inhibition of age-induced miR-195 reverses the senescent phenotype of old MSCs and restores capacity for cell proliferation (Okada et al., 2016). MSC-specific knockout of *Sirt1* in mice results in defects that progress with age, including decline in subcutaneous fat and bone mass, phenotypes which originate from impaired maintenance of osteoblasts and chondrocytes (Simic et al., 2013). Pathology could be rescued by expression of mutant β-catenin that mimics the deacetylated form of β-catenin, strongly suggesting that β-catenin is a Sirt1 target that promotes transcription of genes required for MSC differentiation. SIRT1 is upregulated in osteogenic cultures of bone marrow MSCs and continues to increase progressively with maturation of osteoblasts (Li M. et al., 2018). Oxidative stress can inhibit this increase in SIRT1, along with reduction in alkaline phosphatase activity and mineralization of matrix, two indicators of early and late differentiation, respectively. Yet, pharmacological activation of SIRT1 by resveratrol can protect against oxidative stress and restore mineralization and expression of early- and late-stage differentiation markers (Li M. et al., 2018). Collectively, numerous studies demonstrate roles for SIRT1 in MSCs and their progeny.

SIRT2 appears to have some shared functions with SIRT1, as SIRT2 has also been reported to have an inhibitory effect on adipogenesis of MSCs. SIRT2 deacetylates FOXO1, thus blocking PPARγ expression (Jing et al., 2007; Wang and Tong, 2009; Si et al., 2013). Although SIRT2 is the most abundant of all sirtuins in adipocytes and pre-adipocytes, its mRNA is down-regulated at the initial stages of differentiation in 3T3-L1 cells (Jing et al., 2007). In fact, silencing of *Sirt2* accelerates adipocyte differentiation, evidenced by more rapid accumulation of lipids in *Sirt2* knockdown cells (Jing et al., 2007). Nucleo-cytoplasmic shuttling of SIRT2 regulates its activity and, within the cytosol, SIRT2 is poised to interact with cytosolic and cytoskeletal proteins. For this reason, SIRT2 has been implicated in regulation of cellular morphology through deacetylation of α-tubulin (Inoue et al., 2007; Nahhas et al., 2007). Remodeling of the cytoskeleton via α-tubulin acetylation is crucial for adipocyte development, thus SIRT2 deacetylase activity must be downregulated to permit the cellular reorganization necessary for adipogenesis (Yang et al., 2013).

Three sirtuins, SIRT3, SIRT4, and SIRT5, reside in the mitochondria and regulate the acetylation landscape of mitochondrial proteins. Recent proteomic analyses have also revealed novel modifications targeted by these sirtuins, including succinylation, glutarylation, and malonylation (Carrico et al., 2018). SIRT3 is responsible for deacetylation of the majority of proteins in the mitochondria; whereas, data suggests that SIRT4 and SIRT5 modify low-abundance acetylated proteins or those with other lysine modifications (Lombard et al., 2007). SIRT3 is a major regulator of mitochondrial biogenesis and function in MSCs. Expression of SIRT3 increases in osteoblastic MC3T3-E1 cells (Ding et al., 2017) and over the course of MSC adipogenic differentiation (Hsu et al., 2016). Consistent with positive regulation of differentiation, silencing SIRT3 in MSCs impairs expression of adipo-lineage markers and adiponectin secretion (Hsu et al., 2016). These phenotypic defects are accompanied by decrease in mitochondrial biogenesis and downregulation of PGC-1α, antioxidant enzymes, TFAM, and protein subunits of respiratory enzyme complexes (Hsu et al., 2016). SIRT3 deficiency impairs mitochondrial respiration, inducing a metabolic shift toward glycolysis. SIRT5 modifies the same protein and lysine residue targets as SIRT3 and is the only sirtuin known to catalyze desuccinylation and demalonylation of mitochondrial proteins (Du et al., 2011; Park et al., 2013; Rardin et al., 2013). Unlike SIRT3, SIRT5 expression is reduced during adipogenic differentiation of human MSCs and thus it remains unclear whether SIRT3 and SIRT5 act competitively or synergistically to regulate metabolism (Hsu et al., 2016).

Lastly, SIRT6 and SIRT7 catalyze modification of proteins located in the nucleus and play critical roles in modulating metabolic homeostasis, genome stability, and DNA repair. The majority of research in human MSCs has centered on the roles of these sirtuins in aging. For example, SIRT7 expression declines during human MSC aging, and SIRT7 deficiency accelerates senescence via loss of heterochromatin in regions of innate immune regulation (Bi et al., 2020). In addition, SIRT6 (Chen et al., 2017) and SIRT7 (Cioffi et al., 2015) have been shown to positively regulate adipogenesis. More recent reports using *ex vivo* cultures of MSCs corroborate the importance of SIRT6 in adipocyte maturation from mesenchymal progenitors (Sun et al., 2014; Zhang et al., 2017).

### Mitophagy

Mitochondrial function depends strictly on mitochondrial integrity and quality control. Autophagy of mitochondrial components plays an important role in stem cell maintenance by selective sequestration and degradation of dysfunctional or aged mitochondria. Machinery that participates in clearance of mitochondria is specific to the triggering event, such as differentiation, oxygen imbalance, mitochondrial damage, or, in the context of the germline, removal of paternal mitochondria after fertilization (Pickles et al., 2018). Mitochondrial proteins and other cargo are cleared by the autophagosome. Other quality control mechanisms that contribute to turnover of mitochondria include surveillance by the unfolded protein response, shedding of vesicles, proteolysis, and degradation of mitochondrial proteins by the proteasome (Pickles et al., 2018). Standard culture conditions expose MSCs to excess oxygen (21%) and result in significant increase in mitochondrial ROS production (Phinney et al., 2015). Phinney et al. (2015) found that this oxidative stress induces MSCs to release large vesicles containing entire mitochondria and mitochondrial proteins and structures, such as ATP synthase, OMM, IMM, and cristae. The oxidative stress caused by prolonged exposure to high oxygen induces the Pink1/Parkin-mediated pathway of mitophagy. In addition, a standard marker for autophagosomes, the cytosolic microtubule-associated protein light chain 3 (LC3-I), is found in the mitochondria-containing vesicles, further supporting that mitophagy is stimulated by hyperoxia. Support for a role for mitophagy in differentiation of MSCs is more limited but has been found in the early stages of MSC differentiation, particularly toward the chondrocytic fate (Forni et al., 2016).

Collectively, a body of literature show that differentiation of MSCs is accompanied by and depends upon metabolic reprogramming. Upon fate commitment, several lineages amplify their mitochondrial capacity through replication of mtDNA, increased mitobiogenesis, architectural and morphological reorganization, elevated OXPHOS, and greater ROS generation. Rather than a simple consequence of the differentiation process, these adaptations appear to be necessary for initiation of commitment and lineage maturation. Mitochondrial quality control through autophagy is critical in normal mitochondrial and cellular function. In a broader context, mitochondrial dysfunction is one of the hallmarks of cellular damage and is associated with many human diseases. MSCs hold great potential for use in cell therapies not only because of their multipotency, immunomodulation, and trophic functions but also due to their capacity for mitochondrial transfer to damaged cells. However, the detailed mechanisms and conditions required for efficient mitochondrial transfer between MSCs and recipient cells remain incompletely understood. In the next section, we detail our current understanding of how MSCs transfer mitochondria to regulate metabolism in neighboring cells and their microenvironment.

## Mitochondrial Transfer

Mitochondria can acquire damage as a result of aging, inflammation, injury, genotoxic agents, and oxidative stress. Horizontal transfer of mitochondria or mitochondrial genomes between cells can rescue and rebuild biological function in recipient cells. The first report suggesting that mitochondria could be transported spontaneously between cells described exchange of MitoTracker-positive structures from cardiomyocytes to endothelial cells through transient cytoplasmic bridges or nanotubular structures (Koyanagi et al., 2005). Conclusive evidence for horizontal mitochondrial transfer between cells was published the following year, showing that human bone marrow MSCs could rescue aerobic respiration in cells depleted of mtDNA and mitochondria by ethidium bromide (Spees et al., 2006). In the context of injury and inflammation, MSCs respond to ROS and damage-associated molecular patterns (DAMPs), such as damaged mitochondria and mitochondrial products, by donation of their mitochondria to injured cells (Krysko et al., 2011; Paliwal et al., 2018). Through intercellular mitochondria trafficking, modulation of ROS, and modification of nutrient bioavailability, endogenous MSCs and MSC therapies are believed to exert protective effects by regulation of cellular metabolism in injured tissues. Below, we outline the key players that mediate mitochondrial transfer to cells within damaged tissues from MSCs or stromal derivatives of MSCs. We additionally address therapeutic applications of horizontal mitochondrial transfer by MSCs in regenerative medicine. Other comprehensive reviews are available that address therapeutic applications of mitochondrial transfer for conditions such as mitochondrial-related diseases (Torralba et al., 2016) and the preclinical evidence for the trophic and protective effects of MSC intracellular components (Akyurekli et al., 2015), thus these aspects will not be addressed in detail.

### Transfer Mechanisms

**Figure 2:**
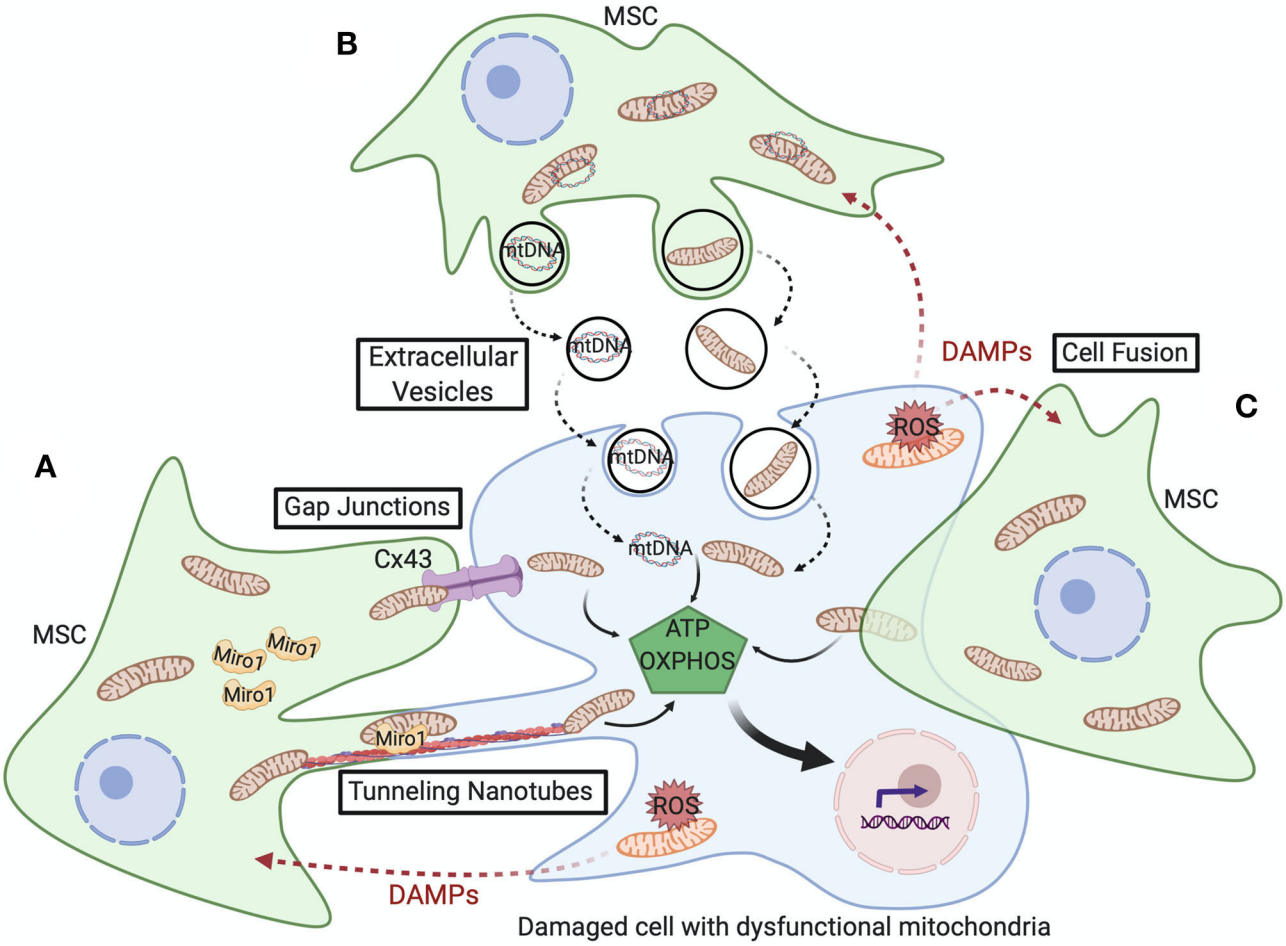
Schematic representation of the various mitochondrial transfer mechanisms utilized between MSCs and damaged cells with dysfunctional mitochondria. **(A)** Tunneling nanotubes (TNTs) are actin-dependent cytoskeletal protrusions that serve as cytoplasmic bridges between cells. Miro1 regulates transport of mitochondria across TNTs. Cx43-mediated gap junctions also serve at cell-cell junctions to enable mitochondrial transfer. **(B)** Extracellular vesicles (EVs) can convey mtDNA or fragments of mitochondria and, though less well-documented, macrovesicles are suggested to contain entire mitochondria. **(C)** Cell fusion enables sharing of cytoplasmic contents during either transient or permanent fusion of the plasma membrane of two cells. Upon transfer of healthy mitochondria from MSCs, respiration increases in the recipient cell and restores cell function, cell survival, and tissue repair.

Cells utilize various mechanisms for transfer of their mitochondrial cargo to other cells, including tunneling nanotubes, gap junctions, extracellular vesicles, and cell fusion. These processes are depicted in Figure 2 and detailed below.

#### Tunneling Nanotubes

Studies of intercellular mitochondrial trafficking are dominated by the role of tunneling nanotubes (TNTs) or membrane nanotubes. TNTs were originally described by (Rustom et al., 2004) as conduits through which mammalian cells can arrange selective transfer of membrane vesicles and organelles between cells. Literature now documents roles for TNTs in exchange of an array of organelles, such as mitochondria, lysosomes, endosomal vesicles, as well as plasma membrane components, lipid droplets, ions, proteins, miRNAs, and pathogens (Gerdes et al., 2007; Davis and Sowinski, 2008). TNT communication allows unidirectional exchange (Rustom et al., 2004; Bukoreshtliev et al., 2009) or bidirectional transfer (He et al., 2011). TNTs form between connecting cells through protrusions of the membrane and have lifetimes ranging from a few minutes to several hours (Gurke et al., 2008). TNTs are ~50–200 nm in diameter and can extend toward neighboring cells at long distances of over 100 μm (Rustom et al., 2004). TNTs contain cytoskeletal filaments composed of F-actin (thin TNT <0.7 μm in diameter) (Rustom et al., 2004) or both F-actin and microtubules (thick TNT > 0.7 μm in diameter) (Önfelt et al., 2006). TNTs containing intermediate filaments and microtubules have also been reported (Resnik et al., 2019). Thin TNTs can transfer portions of plasma membrane between cells, while thick TNTs can carry components of the cytoplasm such as vesicles and organelles, including entire mitochondria. TNT-mediated transfer of mitochondria or mtDNA from MSCs to cells of other organs has been reported in respiratory (Pilling et al., 2006), cardiovascular (Cselenyák et al., 2010), neuronal (Babenko et al., 2015), and immune system (Jackson et al., 2016) diseases and disorders (Table 1).

**Table 1:** Summary of mitochondrial transfer between MSCs and recipient cells of different origins.

**Donor (and model)**	**Recipient**	**Mechanism**	**Cargo**	**Outcome**	**References**
**Respiratory system**					
hBM-MSCs (*in vitro*)	Human alveolar adenocarcinoma cells (mtDNA-depleted A549 cell line)	Cellular contact and Cytoplasmic projections	Mitochondria mtDNA	- ROS and extracellular lactate decreased - ATP production, oxygen consumption, and membrane potential increased - Rescued mitochondrial respiratory function and mtDNA genetic defects	Spees et al., 2006
hBM-MSCs and mBM-MSCs (*in vivo*)	Murine alveolar epithelia cells (Endotoxin-induced damages)	TNTs (Cx43-mediated) and EVs	Mitochondria	- ATP and surfactant production increased - Mitochondrial respiratory function rescued - Animal survival	Islam et al., 2012
BM-MSCs and iPSC-MSCs (*in vitro* and *in vivo*)	Airway epithelial cells (cigarette-smoke-induced damage)	TNTs	Mitochondria	- ATP production and cell bioenergetics increased - Cigarette-smoke-induced alveolar damage reduced	Li et al., 2014
mBM-MSCs (*in vivo*)	Murine lung epithelial cells (Allergen-induced asthma)	TNTs (Miro1-mediated)	Mitochondria	- Inflammation and apoptosis decreased - ATP production and mitochondrial biogenesis increased - Tissue repair and animal survival	Ahmad et al., 2014
BM-MSCs (*in vitro*)	Bronchial epithelial cells	TNTs and EVs	Mitochondria	- Cell survival	Sinclair et al., 2016
iPSC-MSCs (*in vivo*)	Epithelial cells (airway inflammation)	TNTs (Cx43-mediated)	Mitochondria	- Airway inflammation reduced - Mitochondrial biogenetic and function enhanced	Yao et al., 2018
**Immune system**					
hMSCs (*in vitro* and *in vivo*)	Human monocyte-derived macrophages and Murine alveolar macrophages	TNTs and EVs	Mitochondria	- ATP production, mitochondria respiration and bioenergetics increased	Jackson et al., 2016
hMSCs (*in vivo*)	Human monocyte-derived macrophages and murine alveolar macrophages	EVs	Mitochondria	- Inflammatory cytokine secretion decreased - ATP production and mitochondria respiration increased - Macrophage phagocytosis increased	Morrison et al., 2017
**Cardiovascular system**					
MSCs (*in vitro*)	Rat cardiomyoblast (Ischemia-reperfusion induced H9c2 cell line)	TNTs and Cell fusion	Mitochondria	- Cardiomyoblasts bioenergetics preserved	Cselenyák et al., 2010
hBM-MSCs (*in vitro*)	Adult mouse cardiomyocyte	Cell fusion	Mitochondria	- Metabolic reprogramming - Transformation to progenitor state	Acquistapace et al., 2011
hMSCs (*in vitro*)	Human vascular smooth muscle cell (VSMCs)	TNTs	Mitochondria	- MSC proliferation increased	Vallabhaneni et al., 2012
hBM-MSCs (*in vitro*)	Human umbilical vein endothelial cells (HUVECs) (ischemia-reperfusion injury)	TNTs	Mitochondria	- Mitochondria biogenesis increased - Cell apoptosis decreased - Injury rescued and proliferation increased - Cell survival	Liu et al., 2014
BM-MSCs and iPSC-MSCs (*in vivo*)	Mouse cardiomyocytes (doxorubicin-induced damage)	TNTs (Miro1-mediated)	Mitochondria	- ATP production and mitochondria biogenesis increased - Cell viability increased and apoptosis decreased	Zhang et al., 2016
BM-MSCs (*in vitro*)	Rat cardiomyocytes (Ischemic induced H9c2 cell line)	TNTs	Mitochondria	- Mitochondrial function restored - Cell apoptosis decrease	Han et al., 2016
hMADSs (*in vitro* and *in vivo*)	Cardiomyocytes and endothelial cells (with oxidative challenge)	TNTs	Mitochondria	- Mitochondrial biogenesis increased - Cell apoptosis decreased	Mahrouf-Yorgov et al., 2017
**Neurological system**					
BM-MSCs (*in vitro*)	Rat cortical neurons (post-stroke)	Transient cell fusion (Miro1 upregulated)	Mitochondria	- Restored neurological activity and cell survival	Babenko et al., 2015
**Renal system**					
MSCs (*in vitro*)	Rat renal tubular cells	TNTs	Mitochondria	- MSC differentiation into kidney tubular cells	Plotnikov et al., 2010
**Corneal system**					
iPSC-MSCs (*in vitro*)	Corneal epithelial cells	TNTs (Miro1-mediated)	Mitochondria	- Mitochondria respiration and function increased - Corneal wound healing improved	Jiang et al., 2016

*BM-MSCs, Bone marrow mesenchymal stromal cells; hBM-MSCs, Human bone marrow mesenchymal stromal cells; mBM-MSCs, Murine bone marrow mesenchymal stromal cells; iPSC-MSCs, pluripotent stem cell-derived mesenchymal stem cells; hMADS, Human multipotent adipose-derived stem cells; TNT, Tunneling nanotubes; EV, Extracellular vesicles*.

A growing number of studies show that donor cells, including MSCs, must sense cellular damage to exploit TNTs for mitochondrial transport. In human umbilical cord vein endothelial cells exposed to ischemia-reperfusion, TNT generation is dependent upon the ability of MSCs to detect phosphatidylserine (PS) domains on the plasma membrane of the stressed cells (Liu et al., 2014). PS is chiefly oriented toward the cytoplasm on the plasma membrane but moves to the outer leaflet of the membrane (cell surface) during early stages of apoptosis. Masking of PS by annexin V dramatically decreases TNT formation without any significant effect on the quantity of membrane protrusions, indicating that TNT formation by MSCs requires presentation of PS on the membrane protrusions of the injured recipient cell. Other environmental cues and stress conditions, such as hyperglycemia, have also been shown to induce TNT formation in primary cells and cell lines (Lou et al., 2012). In hippocampal astrocytes and neurons, it was proposed that serum starvation and hydrogen peroxide can trigger nanotube formation through p53 activation (Wang et al., 2011). However, using knockout mice and cell lines lacking p53, it has since been shown in murine MSCs and cancer cell lines that TNT development can proceed independently of p53, and, therefore, cells must utilize other stress sensing pathways to signal TNT generation (Andresen et al., 2013).

One mechanism for trafficking of mitochondria within TNTs is mediated by motor-adaptor protein complexes related to the mitochondrial Rho GTPase Miro1 (encoded by the *RHOT1* gene) (Ahmad et al., 2014). Interestingly, mesenchymal cells like smooth muscle cells and fibroblasts express Miro1 and can also donate mitochondria, but they do so with low efficiency relative to MSCs (Ahmad et al., 2014). Indeed, Miro1 is essential for transport of mitochondria, and its overexpression in MSCs was shown to enhance the transfer of mitochondria alveolar epithelial cells in co-cultures. Miro1 overexpression consequently improved rescue of the stressed epithelial cells as measured by ROS accumulation (Ahmad et al., 2014). Indeed, the importance of mitochondrial transfer to the reparative effect of MSCs was also shown in mouse models of lung injury and asthma. MSCs overexpressing Miro1 were more efficient than control MSCs in decreasing airway hyper-responsiveness, decreasing pro-inflammatory cytokines, and restoring ATP levels, and the MSCs did not exhibit changes in their secretory properties or immunomodulatory paracrine signaling. Conversely, Miro1 knockdown prevented TNT formation and caused the loss of MSC efficacy. In experimental stroke, intravenous administration of MSCs overexpressing Miro1 reversed the effects of ischemia and improved recovery of neurological functions better than unmanipulated MSCs (Babenko et al., 2018). Similarly, induced pluripotent stem cell derived (iPSC)-MSCs intrinsically express a high-level of Miro1 and possess higher efficiency of mitochondrial transfer relative to bone marrow MSCs (Zhang et al., 2016). This elevated capacity for mitochondrial trafficking improved MSC efficacy in protection from chemotherapy-induced cardiomyopathy.

A second mechanism for TNT-mediated transport of mitochondria is by gap-junction communication. Connexin 43 (Cx43; encoded by *GJA1*) is a transmembrane protein which can form hemichannels in association with other connexin proteins to enable direct exchange of ions, metabolites, second messengers, microRNAs, and linear peptides between cells or with the extracellular environment (Ribeiro-Rodrigues et al., 2017). Cx43 also mediates TNT formation for mitochondrial transfer through gap junctions between cells (Islam et al., 2012). In a murine model of LPS-induced lung injury, Islam et al. (2012) showed that Cx43 expression on both donor bone marrow MSCs and injured alveolar epithelial cells was required for mitochondrial exchange via TNTs. In this model, TNT establishment was initiated from the cell expressing higher levels of Cx43. Moreover, Cx43 knockdown on MSCs impaired TNT generation and thus these Cx43-deficient MSCs could not rescue the injured epithelial cells (Islam et al., 2012). Further evidence for Cx43 in TNT development in lung injury comes from reports showing that Cx43 is upregulated in mouse lungs during asthma and that overexpression of Cx43 in therapeutically administered iPSC-MSCs restores mitochondrial membrane potential, decreases excess mucus secretion, and reduces inflammatory cells and cytokines caused by lung injury (Yao et al., 2015, 2018). Stable knockdown of Cx43 reduced mitochondrial transfer and abolished the benefits of the cellular therapy.

#### Extracellular Vesicles

Extracellular vesicles (EVs) are heterogeneous subcellular structures consisting of a lipid bilayer, often containing proteins and receptors, that encapsulates molecular cargo present in the cell of origin (Pitt et al., 2016). Depending on their biogenesis, EVs vary in size, morphology, and the contents they transport. Exosomes are EVs created by endocytosis or reverse budding that range in size from 30 to 150 nm in diameter. Exosomes engulf small molecules within the cell, including proteins, lipids, carbohydrates, metabolites, and genetic material including small RNAs, mtDNA, mRNA, and microRNAs, and are subsequently secreted into the extracellular space (Van Niel et al., 2018). On the other hand, microvesicles are EVs created from the outward budding of the plasma membrane to form 0.1–1 μm diameter structures that are able to carry larger cargo such as organelles. Larger microvesicles created by membrane blebbing, such as apoptotic bodies ranging 1–2 μm in diameter and oncosomes 1–10 μm in diameter, can also carry mitochondrial components and other metabolic modifiers (Minciacchi et al., 2015; Jiang et al., 2017).

Exosomes and microvesicles are released by diverse cell types including MSCs (Phinney et al., 2015), platelets, endothelial cells, cancer cells (Raposo and Stoorvogel, 2013), and astrocytes (Hayakawa et al., 2016). MSCs can transfer mtDNA and mitochondrial cargo to macrophages and other cells through release of EVs (Islam et al., 2012; Phinney et al., 2015). Studies have also reported transport of mitochondria via both TNTs and EVs from MSCs to different recipient cells, including lung alveolar epithelial cells and macrophages (Islam et al., 2012; Jackson et al., 2016). Compared to TNTs, the mechanisms mediating mitochondrial transfer by EVs are less well-understood. Additionally, evidence suggests that compromised mitochondria might be preferentially exported in this context. Phinney et al. (2015) found that human MSCs target depolarized mitochondria for packaging at the plasma membrane via arrestin domain-containing protein 1-mediated microvesicles. The mitochondria containing vesicles are engulfed and reutilized by macrophages. Interestingly, the macrophages are simultaneously subjected to de-sensitization by miRNA-containing exosomes produced by the MSCs that inhibit activation of Toll-like receptor signaling and prevent rejection of the ingested mitochondria. Despite the poor quality of the donor mitochondria, the recipient macrophages demonstrate greater bioenergetic capacity, suggesting mutual benefit to both the MSCs and macrophages.

#### Cell Fusion

Cell fusion is a process in which uninuclear cells combine to share organelles and cytosolic components by merging plasma membranes and forming multinuclear cells. Cell fusion can be partial (temporary) or complete (permanent). Partial cell fusion includes a direct transient exchange of protein complexes and subcellular organelles between cells. A complete cell fusion occurs when cells share the entire cytoplasm and acquire a unique permeant karyotype. Cell fusion is predominantly reported to modulate the development of organs during embryogenesis, morphogenesis, and cellular differentiation. Indeed, the placenta, bone, and muscle rely on cell fusion for proper organ function.

Cell fusion is used, albeit rarely, for MSC-driven mitochondria sharing. Underlying mechanisms for mitochondria exchange through cell fusion remain elusive; although, rearrangement of the actin cytoskeleton and fusogenic glycoproteins across the membranes of both cells is required (Aguilar et al., 2013; Shilagardi et al., 2013). Mitochondria transfer has been shown to occur through complete cell fusion after the selective loss of the donor cell's nucleus (Alvarez-Dolado et al., 2003). Acquistapace et al. (2011) reported that mitochondrial transfer via partial cell fusion of human bone marrow MSCs or human multipotent adipose-derived stem cells (hMADS) with mature murine cardiomyocytes could promote reprogramming of the cardiomyocytes to a progenitor-like state. In this process, mtDNA depletion of MSCs did not affect cell fusion capacity, but it did result in a dramatic decrease in their reprogramming efficiency. These data suggest that de-differentiation of the cardiomyocytes was due to metabolic reprogramming by MSC mitochondria (Acquistapace et al., 2011).

### Therapeutic Applications in Mitochondrial Transfer

Accumulating data implicates mitochondrial donation from MSCs as a critical component of their therapeutic efficacy. Several diseases and organ systems have been examined, including the respiratory, cardiovascular, neurological and renal systems. Table 1 summarizes representative reports from the current literature.

#### Neurological System

Several ongoing clinical trials indicate the feasibility and safety of using MSCs for the treatment of neurological injury and degenerative diseases affecting the brain, spinal cord, and visual system. Whereas, some studies implicate the neuroprotective secretome of MSCs in fostering repair and regeneration, others suggest that metabolic rescue is crucial for the therapeutic benefit of MSCs. Babenko et al. (2015) demonstrated that preferential cytosol transfer from neural cells to MSCs could enhance their neuroprotective capacity. This priming from coculture with neurons resulted in doubling of Miro1 abundance and elevated production of brain-derived neurotrophic factor in MSCs. Mitochondrial transfer was confirmed from MSCs to neurons and astrocytes in cocultures. Using this priming method in a stroke injury model, authors showed that delivery of neuron-primed MSCs reduced total volume of ischemic lesions and severity of neurological deficits, as measured by response to tactile and proprioceptive stimuli (Babenko et al., 2015). Retinal ischemia caused by stroke can also cause lifelong visual impairments attributed to mitochondrial dysfunction and cell death of the retinal ganglion cells and pigmented epithelium. Nguyen et al. (2019) showed that intravenous transplantation of MSCs restores mitochondrial respiration, morphology, and dynamics in these cells, suggesting that MSC therapy after stroke could also benefit the visual system through mitochondrial transfer. Retinal ganglion cell repair by MSC mitochondrial transfer was also shown in a model of retinal degeneration in which mice lacking the NADH dehydrogenase Fe-S protein 4 gene (*Ndufs4*) experience deterioration of the nerves of the eyes (Jiang et al., 2019). Following local delivery of human iPSC-MSCs into the vitreous cavity of the eye, successful transfer of mitochondria was confirmed in retinal ganglion cells and their loss by cell death was prevented. Electrical activity measured by electroretinogram also indicated that MSCs protected visual function. Authors confirmed absence of human nuclear DNA in the mouse retina, arguing against permanent cell fusion or direct differentiation of MSCs into neural cell lineages.

#### Respiratory System

The therapeutic effect of mitochondrial transfer from MSCs has been demonstrated in respiratory disease by several independent groups using multiple models of lung injury and asthma. Islam et al. (2012) were the first to report that it was mitochondrial transfer from MSCs to pulmonary alveoli that restored alveolar bioenergetics. They showed that intranasal or intratracheal instillation of human or murine MSCs increased animal survival in lipopolysaccharide (LPS)-induced acute lung injury. Using live lung microscopy, they observed that mitochondrial transfer occurred through Cx43-driven TNTs and by EVs in a Ca^2+^ dependent manner. MSCs reduced leukocyte numbers, normalized surfactant secretion, and improved ATP levels in alveolar epithelia. In an allergen-induced asthma model, Ahmad et al. (2014) demonstrated that MSC efficacy and TNT-mediated mitochondrial transfer from MSCs to injured lung epithelial cells could be amplified by overexpression of Miro1 in donor MSCs, while its knockdown reduced therapeutic efficacy. In this study, instillation of MSCs reduced apoptosis in airway epithelium and rescued mitochondrial biogenetics. In a subsequent asthma mouse model study, local transplantation of iPSC-MSCs reduced airway inflammation and improved mitochondrial function in damaged epithelial cells through Cx43-dependent TNTs (Yao et al., 2018). Additionally, in a model of chronic obstructive pulmonary disease, mitochondrial transfer from iPSC-MSCs was shown to be more effective than bone marrow MSCs in rescuing cigarette smoke-induced mitochondrial dysfunction, attributed in part to greater mitochondrial transfer efficiency from the pluripotent source of MSCs (Li X. et al., 2018).

#### Immune System

The therapeutic properties of MSCs derive in part from their capacity to modulate the innate and adaptive immune systems, including macrophages, dendritic cells, NK cells, and T and B lymphocytes (Weiss and Dahlke, 2019). *Ex vivo* and *in vivo* studies have demonstrated that mitochondrial transfer from MSCs to macrophages improves phagocytic activity in the recipient cells (Jackson et al., 2016). Phinney et al. (2015) showed that under oxidative stress, MSCs undergo mitophagy and unload their depolarized mitochondria to macrophages. The EVs engulfed by macrophages augmented macrophage bioenergetics. In a study of acute respiratory distress syndrome, EV-mediated mitochondrial transfer from MSCs to macrophages resulted in repression of proinflammatory cytokine secretion and improved phagocytic capacity by promoting an M2 anti-inflammatory phenotype (Morrison et al., 2017). Collectively, these studies aid our understanding of how metabolic reprogramming can modify the inflammatory profiles of immune cells and complement the direct production of immunomodulatory factors by MSCs.

#### Cardiovascular System

Mitochondrial dysfunction is implicated in the development and pathophysiology of heart failure (Zhou and Tian, 2018). As in the lung, mitochondrial transfer in cardiac tissue has been shown to improve outcomes through metabolic reprogramming (Koyanagi et al., 2005; Plotnikov et al., 2008). In an *ex vivo* ischemic heart disease model, bone marrow MSCs rescued damaged cardiomyocytes through TNT-mediated mitochondrial transfer (Han et al., 2016). In this study, mitochondrial membrane potential and function were elevated in the cardiomyocytes and apoptosis was reduced (Han et al., 2016). Further, MSCs with a high efficiency of mitochondrial transfer, iPSC-MSCs expressing Miro1 at high intrinsic levels and those with ectopic Miro1 expression, were more effective in alleviating injury to cardiac cells in a model of anthracycline-induced cardiomyopathy (Zhang et al., 2016). In co-cultures of bone marrow MSCs and terminally differentiated mouse cardiomyocytes, partial cell fusion and the resulting donation of mitochondria promoted reversion of mature cardiomyocytes to a progenitor-like state (Acquistapace et al., 2011).

Multiple studies have also reported bidirectional trafficking of mitochondria. For example, in co-cultures of human primary MSCs and vascular smooth muscle cells (VSMCs), no preferential direction of mitochondrial movement was observed between the cells (Vallabhaneni et al., 2012). Moreover, the mitochondrial transfer from VSMCs to MSCs promoted proliferation of the recipient MSCs (Vallabhaneni et al., 2012). Additionally, mtDNA-depleted VSMCs were unable to stimulate MSC proliferation, indicating that functional mitochondria in the donor cells was instrumental in initiating cell cycling. Mahrouf-Yorgov et al. (2017) reported that damage signals released by injured cells can activate anti-apoptotic and regenerative functions of MSCs. In *in vitro* models of hydrogen peroxide or doxorubicin-induced cellular damage and a model of myocardial infarction, they found that MSCs sense and uptake DAMPs, including dysfunctional mitochondria, that are released from endothelial cells or cardiomyocytes (Mahrouf-Yorgov et al., 2017). MSCs engrafted into infarcted hearts upregulated heme oxygenase-1 and increased mitochondrial biogenesis. Conversely, when phagocytosis or heme oxygenase activity was inhibited, engrafted MSCs lost their ability to protect cardiac tissue. Collectively, these data highlight the role that mitochondria play as signaling molecules and the importance of MSC sensing of the microenvironment to trigger therapeutic efficacy.

In summary, mitochondrial transfer plays a central role in metabolic rescue and phenotypic alteration of a variety of organ systems that are responsive to MSC therapy. Multiple lines of evidence support the importance of bi-directional signaling between donor and recipient for activation of MSC trophic and immune regulatory activities. This crosstalk can occur via engulfment of mitochondrial cargo and other DAMPs by MSCs, sharing of cytosolic components, and/or interactions at the plasma membrane. MSCs are then primed to donate mitochondria to neighboring cells in need to assist in restoration of damaged tissue.

## MSCs as Modifiers of Cancer Metabolism

These same mechanisms that are beneficial in regenerative medicine can be hijacked in malignancy, whereby transfer of mitochondria and/or mtDNA to cancer cells increases mitochondrial content and enhances OXPHOS to favor proliferation and invasion (Herst et al., 2018). Cancer cell survival, proliferation, and motility often requires adaptation to oxygen-poor conditions and increased nutrient demand. Cancer cells use multiple metabolic strategies to overcome these challenges and obtain the resources they need for ATP production and anabolic processes (Luengo et al., 2017). Some of these strategies include altered expression of metabolic genes or utilization of alternate fuels, but others include exploitation of the metabolic capacities of non-malignant cells associated with the tumor.

MSCs contribute to maintenance of normal tissue homeostasis, but also serve as a reservoir for generation of other stromal derivatives, such as cancer associated fibroblasts (CAFs) and tumor-associated stroma, that alter the biophysical, chemical, and cellular composition of the tumor microenvironment. MSCs recruited to tumors can differentiate into CAFs or remain as multipotent tumor-associated MSCs (Liu et al., 2019). Collectively, the stromal components of the tumor microenvironment have been linked to tumorigenesis, angiogenesis, metastasis, immunosuppression, drug resistance, maintenance of cancer stemness, extracellular matrix remodeling, and metabolic reprogramming (Liu et al., 2019). The role of MSCs in tumor initiation, growth, and resistance to treatment is debated, but their ability to modify cancer cell metabolism and the metabolic environment suggests that MSCs are centrally poised to alter malignancy, angiogenesis, and immune cell infiltrates. We detail below the roles that MSCs play in shaping the metabolic landscape in cancer.

### Solid Tumors

MSCs or other MSC derivatives have been shown to donate mitochondria to cell lines from breast cancer, prostate cancer, lung adenocarcinoma, melanoma, glioma, and osteosarcoma (Spees et al., 2006; Schichor et al., 2012; Pasquier et al., 2013; Caicedo et al., 2015; Lin et al., 2015; Dong et al., 2017; Ippolito et al., 2019). The first demonstration of mitochondrial transfer from MSCs was observed during the co-culture of bone marrow MSCs and human A549 lung adenocarcinoma cells depleted of mtDNA by serial passage with ethidium bromide (ρ° or rho-zero cells) (Spees et al., 2006). Further, it was shown that the respiratory defects of ρ° cells are rescued upon receipt of mitochondria or mtDNA from MSCs or even by uptake of isolated mitochondria or mtDNA available in the medium (Spees et al., 2006; Caicedo et al., 2015; Pacak et al., 2015). In two more recent studies, one group showed that ρ° B16 mouse melanoma and ρ° 4T1 breast carcinoma cells were able to form tumors *in vivo* only when they acquired mitochondria (and mtDNA) from host cells at the transplant site (Tan et al., 2015; Dong et al., 2017). A fully functional respirasome (CI, CII, and CIV supercomplex) was restored in B16 cells of the primary tumor and in metastases, with evidence of mtDNA from the host shown by sequencing. Indeed, ρ° cells recovered from tumors exhibited similar levels of respiration as the parental lines. This was confirmed through measurement of lactate and ATP levels; glucose uptake; and enzymatic activity of succinate dehydrogenase, succinate quinone reductase, and citrate synthase. In independent studies, transfer of mitochondria to *in vitro* cultured cancer cells has been found to result in elevated levels of OXPHOS and greater tumorigenicity, proliferation, and invasiveness (Caicedo et al., 2015; Lu et al., 2017). Mitochondrial transfer to breast cancer cells was also shown to enhance chemoresistance to doxorubicin (Pasquier et al., 2013). Collectively, these data suggest that mitochondrial transfer from stroma can play a key role in facilitating bioenergetic resilience of cancer cells *in vitro* and within the tumor microenvironment.

Reports of mitochondrial transfer from MSCs and CAFs to cancer cells point to TNTs as the chief delivery route (Pasquier et al., 2013; Ippolito et al., 2019). Yet, coupling between MSCs and glioma cells has also shown that MSCs share mitochondrial content via Cx43 gap junctions and cell fusion, which authors described as syncytia because of the extensive structural interactions and functional exchange of cellular contents (Schichor et al., 2012). Future research should aim to determine the effects of metabolic modification of tumors by the stroma on drug resistance mechanisms and immunogenicity of cold tumors (Liu and Curran, 2020).

### Hematological Malignancies

Bone marrow niches, consisting of various cell types including stromal and hematopoietic cells, have critical roles in regulating the fate of adult HSCs in terms of quiescence, self-renewal, and differentiation (Méndez-Ferrer et al., 2020). These niches maintain hematopoietic homeostasis through soluble factors, direct cell-cell contact, and cell-surface ligands. Indeed, MSCs directly interact with HSCs and secrete hematopoietic cytokines and growth factors that dictate HSC cell cycle entry and lineage maturation (Saleh et al., 2015). In hematological malignancies, remodeling of bone marrow niches has been found to create an environment that supports malignant stem cells and impairs maintenance of normal HSCs (Pievani et al., 2020). Crosstalk with cells in the bone marrow microenvironment can also contribute to drug resistance and relapse via modification of gradients of soluble factors/metabolites, dysregulation of extracellular matrix deposition, and by direct cell-cell interactions, many of which alter cancer cell metabolism (Konopleva et al., 2002; Samudio et al., 2008). Cell intrinsic mutations can also drive these metabolic adaptations (Faubert et al., 2020). Moreover, it is clear that these strategies of adaptation to nutrients in the environment and metabolic reprogramming are frequently employed by hematological malignancies to support aggressive anabolic growth and chemoresistance (Basak and Banerjee, 2015). Table 2 summarizes reports of mitochondrial transfer in hematological malignancies.

**Table 2:** Summary of mitochondria transfer between MSCs and recipient cells of hematological malignancies.

**Recipient**	**Model**	**Mechanism**	**Outcome**	**References**
**Acute myeloid leukemia (AML)**				
Primary AML blasts and AML cell lines: HL-60, Kasumi-1, KG-1, MOLM-14, NB-4, SKM-1, THP-1, and U-937	*In vitro* coculture and NSG xenograft model	TNT-mediated and endocytosis-dependent mitochondrial transfer to AML cells	- ATP and OXPHOS production increased - Increased tumorigenic potential and chemoresistance	Moschoi et al., 2016
Primary AML blasts	*In vitro* coculture and NSG xenograft model	TNT-mediated mitochondrial transfer to AML cells upon NOX2 upregulation and ROS enhancement	- Extracellular lactate production reduced - ATP level, oxygen consumption and mitochondrial membrane potential increased - NOX2 inhibition blocked mitochondrial transfer, increased AML cell apoptosis, and improved animal survival in xenotransplantation	Marlein et al., 2017
**Acute lymphocytic leukemia (ALL)**				
Primary B-ALL blasts and BCP-ALL cell lines: NALM6 (B-Other) and REH (TEL-AML1)	*In vitro* coculture	TNT-mediated mitochondrial transfer to BCP-ALL cells	- Increased tumorigenic potential and chemoresistance	Polak et al., 2015
Primary T-ALL cells and T-ALL cell line: Jurkat	*In vitro* coculture	Not described	- Glucose uptake and lactate production increased in T-ALL cells - Mitochondrial membrane potential and ROS level decreased - Promoted a pro-glycolytic shift in T-ALL cells - Mitochondrial fission governed by ERK-mediated phosphorylation of Drp1 - T-ALL cell chemoresistance	Cai et al., 2016
B-precursor ALL cell lines: REH, SD1, SEM, and TOM1	*In vitro* coculture and NSG xenograft model	TNT-mediated mitochondrial transfer to BCP-ALL cells	- MSC acquisition of cancer-associated fibroblast phenotype - BCP-ALL cell chemoresistance - Tumor progression in animals - Microtubule inhibitors interrupted mitochondrial trafficking and reduced drug resistance	Burt et al., 2019
Primary T-ALL cells and T-ALL cell line: Jurkat	*In vitro* coculture	Bidirectional TNT-mediated mitochondrial transfer modulated by adhesion molecule ICAM-1	- T-ALL cells transfer more mitochondria to MSCs while receive fewer - T-ALL cells chemoresistance - Blockage of mitochondria transfer by inhibiting TNT formation decreased the drug cytotoxicity of T-ALL cells	Wang et al., 2018
**Multiple myeloma (MM)**				
Primary MM cells and MM cell lines: MM1S and U266	*In vitro* coculture and NSG xenograft model	TNT-mediated mitochondrial transfer mediated by CD38	- ATP level and OXPHOS increased in MM cells - Chemotherapy drugs increased mitochondrial transfer and MM cell tumorgenicity - CD38 knockdown blocked mitochondrial transfer and improved animal survival	Marlein et al., 2019

#### Acute Myeloid Leukemia

Substantial evidence points to the bone marrow microenvironment as a major contributor to the pathogenesis of leukemia, including adaptations in energy metabolism that confer resistance to chemotherapy. Bone marrow MSCs have been shown to drive reprogramming of acute myeloid leukemia (AML) metabolism toward aerobic glycolysis thereby promoting the Warburg effect by release of paracrine effectors (Samudio et al., 2008). In this study, MSCs induced depolarization of the mitochondria of AML cells by upregulation of uncoupling protein 2 (UCP2), leading to decreased production of ROS and reduced susceptibility to chemotherapeutic drugs, including mitoxantrone, cytosine arabinoside, and vincristine. Another mechanism proposed to protect AML cells from toxic ROS accumulation is export of superoxides to MSCs through gap junctions, as has been demonstrated to protect HSCs in the aging niche (Ishikawa et al., 2012).

As observed in solid tumors, mitochondrial transfer from MSCs is also reported in hematological malignancies, including AML. Primary and cultured AML cells readily uptake mitochondria from bone marrow stromal cells both *in vitro* and *in vivo* in xenotransplantation models (Moschoi et al., 2016; Marlein et al., 2017). Compared to HSCs, AML cells contain more mtDNA, larger numbers of mitochondria, and depend heavily on OXPHOS (Boultwood et al., 1996; Škrtić et al., 2011). Bone marrow stroma serve as an important reservoir of mitochondria for AML cells, as elevated OXPHOS can only be maintained in culture with MSCs containing functional mitochondria. AML cells cultured with ρ° MS-5 stromal cells or separated from MS-5 cells by transwell membranes fail to maintain high levels of mitochondrial ATP production (Moschoi et al., 2016). Marlein et al. (2017) showed that AML initiates mitochondrial transfer with MSCs by NADPH oxidase-2 (NOX2)-dependent superoxide production. This mechanism not only improves bioenergetics in AML cells, but also increases chemoresistance to cytarabine (Moschoi et al., 2016). In this way, AML cells exploit oxidative stress to acquire greater mitochondrial capacity and chemoresistance. Indeed, some commonly used chemotherapeutic agents amplify mitochondrial transfer to AML, including cytarabine, etoposide, and doxorubicin (Moschoi et al., 2016). Thus, targeting mitochondria and/or mitochondrial transfer could have therapeutic advantages in chemotherapy-resistant AML (Lagadinou et al., 2013). For example, cytarabine residual AML cells (cytarabine resistant) utilize high levels of OXPHOS and thus agents that inhibit mitochondrial protein synthesis, electron transfer, or fatty-acid oxidation have the greatest anti-leukemic effects in this resistant population (Farge et al., 2017).

Several of the approaches used to disrupt mitochondrial transfer are aimed at blocking TNTs, endocytosis, or superoxides that can prime MSCs. The CD38 surface marker has been implicated in TNT formation and mitochondrial transfer from MSCs to AML, but also can be leveraged for targeting AML cells for phagocytosis by anti-CD38 antibodies (Farber et al., 2018; Mistry et al., 2019). Daratumumab is the first of several anti-CD38 monoclonal antibody-based therapeutics that improve anti-tumor immunity and also demonstrate an inhibitory effect on AML mitochondrial transfer *in vitro* and *in vivo* (Farber et al., 2018; Mistry et al., 2019). Master regulators of mitochondrial biogenesis and activity including PGC-1α and NOX2 are also promising targets. AML blasts stimulate MSCs to produce more mitochondria, consume more oxygen, and activate PGC-1α (Marlein et al., 2018). PGC-1α was important for mitochondrial transfer to AML cells, and authors showed that inactivation of PGC-1α by knockdown or by reduction in superoxide levels with N-acetylcysteine impaired mitochondrial transfer (Marlein et al., 2017, 2018). In an NSG xenograft model, PGC-1α deficient bone marrow stromal cells co-transplanted subcutaneously with AML cells produced smaller tumors relative to control stroma (Marlein et al., 2018). Similarly, inhibition of NOX2 in NSG mice increased AML apoptosis and improved animal survival by preventing mitochondrial transfer (Marlein et al., 2017). Together, these studies highlight the importance of not only exploiting metabolic dependencies of the tumor cell but also understanding how the cells within the microenvironment enable bioenergetic resilience and chemoresistance of the leukemia cells.

#### Acute Lymphoblastic Leukemia

Acute lymphoblastic leukemia (ALL) is another hematological malignancy that disrupts normal HSC and progenitor niches as they overtake the local microenvironment of the bone marrow (Colmone et al., 2008). TNT-mediated mitochondrial transfer between B-cell precursor ALL (BCP-ALL) cells and surrounding MSCs has been reported to be a mechanism by which ALL cells regulate release of cytokines and chemokines from the cells that comprise the HSC niche (Polak et al., 2015). Authors showed that BCP-ALL cells use TNTs to signal to primary MSCs. This communication stimulates modification of niche factors, including pro-survival cytokines and chemokines, that promote BCP-ALL survival and chemotherapy resistance. Importantly, disruption of TNTs resensitizes BCP-ALL to treatment. Burt et al. (2019) also suggest that chemotherapy resistance and relapse in ALL arises not from a specific mutation in ALL cells but rather from sheltering of ALL cells by a niche in the bone marrow that protects from the toxic ROS accumulation that many chemotherapeutics rely upon for killing. They made the novel observation that MSCs isolated from the bone marrow of patients treated for ALL with standard induction chemotherapy and rituximab had an abundance of activated MSCs with a CAF-like phenotype. To test whether MSC activation provided protection for ALL cells from chemosensitivity, they modeled MSC activation *in vitro* with ROS-inducing agents and observed that ALL cells experienced significantly less cell death when able to engage in direct cell-cell contact with MSCs. They further found that toxic levels of intracellular ROS were reduced in ALL cells exposed to chemotherapy, which was mediated by TNT-based communication and transfer of mitochondria from MSCs. Blocking mitochondrial transfer by mitochondrial depletion of MSCs, microtubule inhibitors, or co-culture in transwells impaired ALL cell survival. In another study, bidirectional TNT-mediated mitochondrial transfer between T cell acute lymphoblastic leukemia (T-ALL) cells and surrounding MSCs was also found to contribute to chemoresistance of this hematological malignancy (Wang et al., 2018). During this interaction, which was mediated by the adhesion molecule ICAM-1, T-ALL cells transferred more mitochondria to MSCs while receiving fewer from MSCs (Wang et al., 2018). In this model, increased mitochondria transfer from T-ALL cells to MSCs was reported as the mechanism employed by T-ALL cells to survive the chemotherapy-induced intracellular ROS (Wang et al., 2018). The preference for export of mitochondria from T-ALL cells was suggested to be due to a bias toward glycolytic ATP production in contrast to reliance on OXPHOS in AML (Suganuma et al., 2010). Nevertheless, these reports suggest that blocking the route of mitochondria trafficking between MSCs and hematological malignancies is an important area for future research that could have promising therapeutic potential in relapsed or refractory tumors.

#### Multiple Myeloma

Similar to T-ALL, heavy reliance on glycolysis is observed in a large subset of patients with multiple myeloma (MM) (Borsi et al., 2014; Dalva-Aydemir et al., 2015). Yet, inhibition of glucose metabolism can lead to compensatory engagement of glutamine oxidation that enables survival and chemoresistance of MM cells (Adekola et al., 2015; Marlein et al., 2019). Marlein et al. (2019) reports that mitochondrial transfer from MSCs to MM cells via tumor-derived TNTs promotes this metabolic switch to OXPHOS. Inhibition of endocytosis by dansylcadavarine had no impact on mitochondrial transfer; whereas, the inhibitor of actin polymerization cytochalasin B significantly reduced mitochondrial transfer. TNT formation was observed in ~60% of MM cells in co-culture with bone marrow MSCs, and residual lipid membrane from MM cells was prevalent on MSCs, suggesting that MM readily establish TNTs with MSCs. Mitochondrial trafficking was prevented by a blocking antibody against or knockdown of the surface molecule CD38, which also reduced tumor burden and improved animal survival. Agents targeting CD38 have attracted attention for their inhibitory effect on mitochondrial trafficking between hematologic cancers and MSCs. For example, treatment of relapsed or refractory MM by daratumumab or isatuximab show marked improvements in overall response rate and progression-free survival (Krejcik et al., 2016; Martin et al., 2019; Moreno et al., 2019). Mitochondrial transfer therefore should be considered part of the malignant phenotype of MM and one that serves to facilitate chemoresistance. Altogether, targeting TNT-mediated communication between hematologic malignancies and their supportive niche offers a promising approach to overcome drug resistance and relapse.

## Conclusions

Mitochondria and regulators of mitochondrial activity have emerged as critical determinants of MSC differentiation and function. The contribution of MSCs to homeostasis of hematopoietic stem and progenitor cells within the bone marrow is well-accepted. Yet, as clinical applications for MSC therapies expand, we are now learning that a critical component of their regenerative and tropic effects includes donation of their mitochondria to damaged cells. This same altruistic behavior of MSCs can also exacerbate cancer progression and confer chemoresistance to solid and hematological cancers. Relapse after chemotherapy continues to be a major challenge, thus demand for new therapeutic strategies that explore interruption of mitochondrial transfer and metabolic regulation by MSCs is high. Given that intercellular mitochondrial shuttling has broad implications for treatment of injury and disease, further investigation of the mechanisms that regulate MSC priming and mitochondrial transfer should be a future priority.

## Author Contributions

AM and PW contributed to conception and design of the review outlines. AM prepared the figures and tables. All authors contributed to writing sections of the text, revision, reading, and approval of the submitted manuscript.

## Conflict of Interest

The authors declare that the research was conducted in the absence of any commercial or financial relationships that could be construed as a potential conflict of interest.
